# Supplementary material for: Nucleation of protein mesocrystals via oriented attachment
Source: Nat Commun. 2021 Jun 23;12:3902. doi: 10.1038/s41467-021-24171-z (PMC8222410; doi:10.1038/s41467-021-24171-z)
Supplement: Supplementary file 1 — Supplementary Information [file 41467_2021_24171_MOESM1_ESM.pdf]

Supplementary Information to

Nucleation of protein crystals via oriented attachment

Alexander E.S. Van Driessche[1], Nani Van Gerven[2,3], Rick R.M. Joosten[4,5], Wai Li Ling[6], Maria Bacia[6], Nico A.J.M. Sommerdijk[7], and Mike Sleutel[2,3*]

[1] Univ. Grenoble Alpes, CNRS, ISTerre, F-38000 Grenoble, France

[2] Structural Biology Brussels, Vrije Universiteit Brussel, Pleinlaan 2, 1050 Brussels, Belgium

[3] Structural and Molecular Microbiology, Structural Biology Research Center, VIB, Pleinlaan 2, 1050 Brussels, Belgium

[4] Laboratory of Materials and Interface Chemistry and Center of Multiscale Electron Microscopy, Department of Chemical Engineering and Chemistry, Eindhoven University of Technology, PO box 513, 5600MB Eindhoven, The Netherlands.

[5] Institute for Complex Molecular Systems, Eindhoven University of Technology, PO box 513, 5600MB Eindhoven, The Netherlands

[6] Univ. Grenoble Alpes, CEA, CNRS, IRIG, IBS, 38000 Grenoble, France

[7] Department of Biochemistry, Radboud Institute of Molecular Life Sciences, Radboud University Medical Center, Geert Grooteplein 6525 GA Nijmegen, The Netherlands[4] Univ. Grenoble Alpes, CEA, CNRS, IRIG, IBS, 38000 Grenoble, France

[*]Correspondence to: mike.sleutel@vub.be

Contents:

Supplementary Figures: 7

Supplementary Table: 2

Supplementary Note

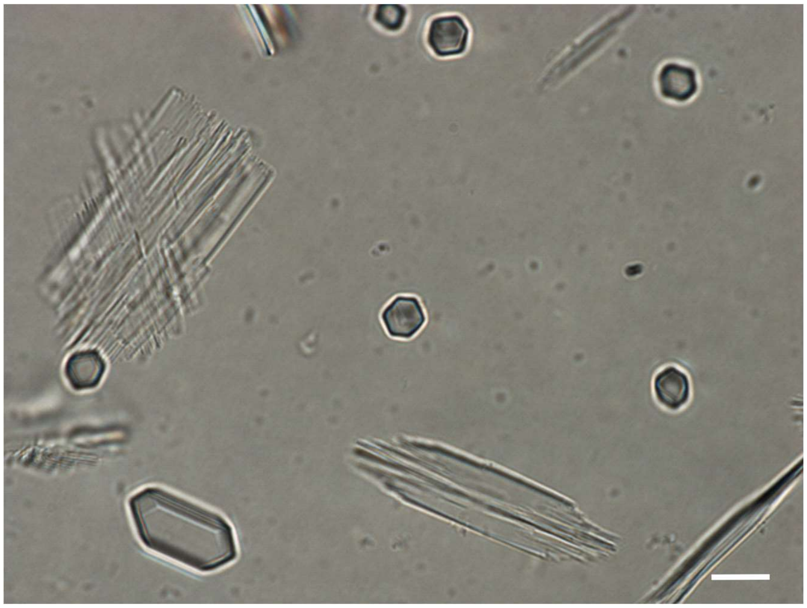

Supplementary Figure 1. GI R387A polymorphism: Polarized light microscopy image of two GI R387A polymorphs (rhombic: I222; twinned platelets: H32) formed 10 minutes after mixing protein stock solution with PEG-1000 (10min, Scalebar: 5µm).

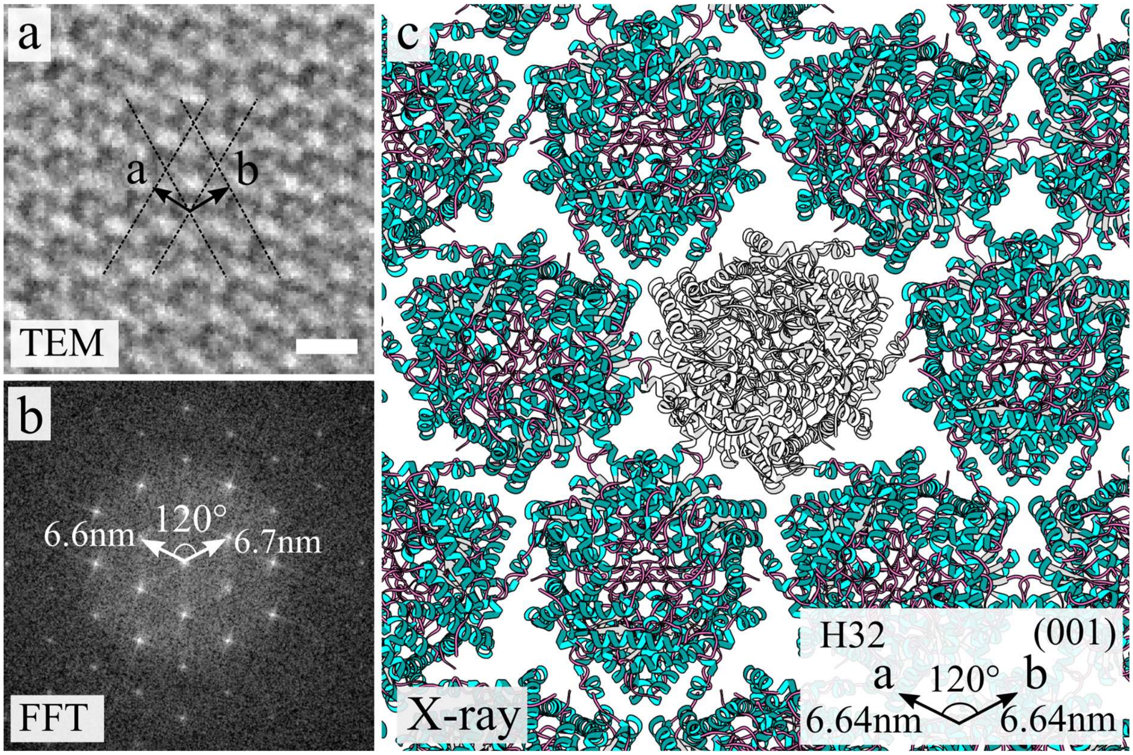

Supplementary Figure 2. Crystallographic arrangement of GI molecules in the H32 space group: (a) Raw cryoEM image of the lattice of R387A GI nanocrystals formed in 15mg.mL^{-1} GI, 50mM Hepes pH 7.0, 100mM $MgCl_2$, 4% (w/v) PEG-1000. Scalebar is 10nm; (b) FFT of panel (a) with indicated nearest neighbor distances and measured angle; (c) crystallographic arrangement of GI molecules along the (001) plane of the trigonal H32 space group (as determined by X-ray diffraction of S171W crystals) with the theoretical nearest neighbor distances along the a and b axis and corresponding angle (inset).

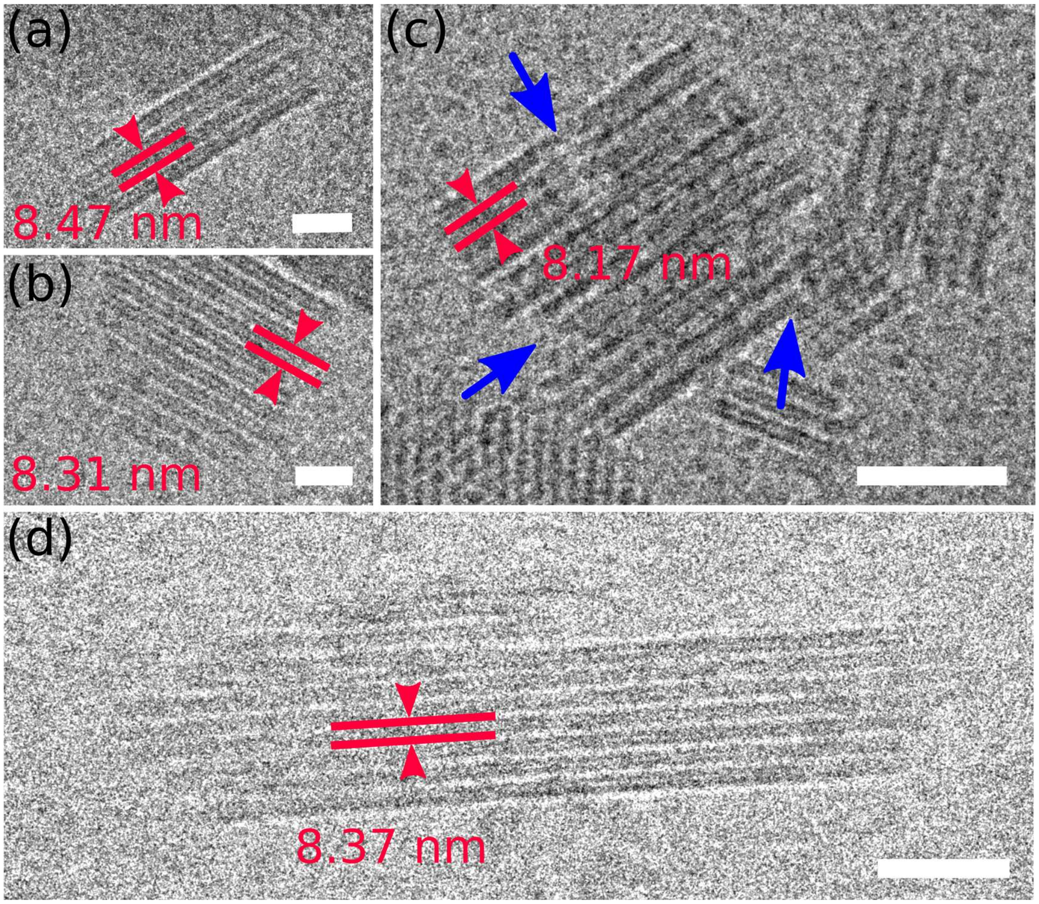

Supplementary Figure 3. Side-view of submicron H32 nanocrystals: Side-view images of submicron H32 nanocrystals formed 1min40s after mixing GI R387A with PEG 1000, with lattice distances denoted in red. The smaller crystallites next to the bigger crystal cluster in (c), as well as the local discontinuities highlighted by the blue arrows are also suggestive of OA mediated crystallite stacking along the c-axis. Scalebar in (a) and (b) is 25nm, and in (c) and (d) 50nm.

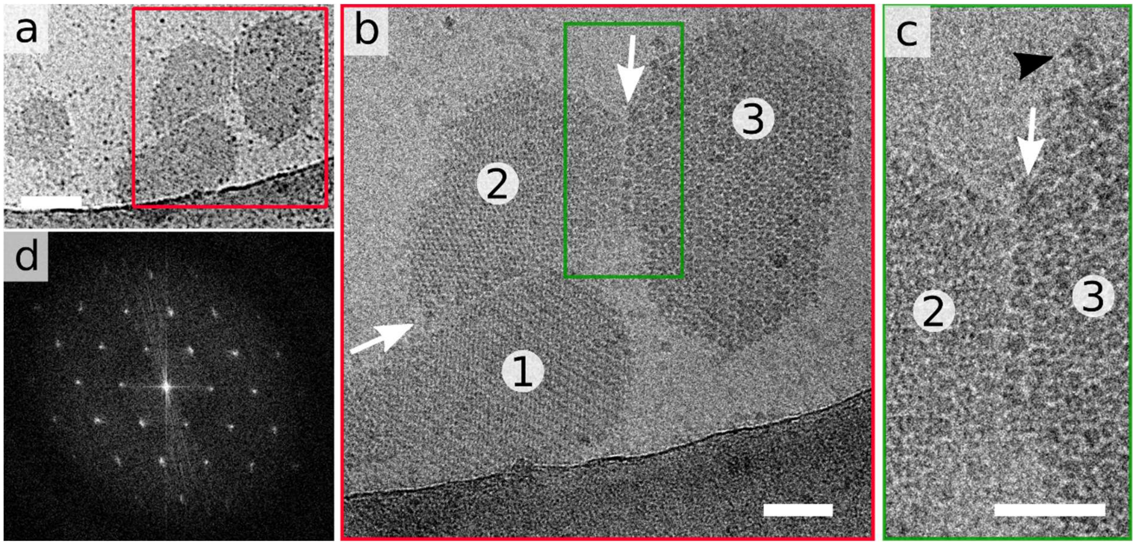

Supplementary Figure 4. Oriented attachment of R387A GI nanocrystals: Additional example of OA of 3 R387A GI nanocrystals: low magnification image (a) with consecutive zoom-ins of the local cluster (b) and the binding interface (white arrow) between 2 and 3 encompassing approximately 8 GI molecules (c). Black arrowhead: incoming GI tetramer; the sharp maxima in the FFT of the highlighted area (red) demonstrates good alignment of the respective lattices (d). Scalebar in (a) is 100nm, in (b) 50nm and in (c) 25nm.

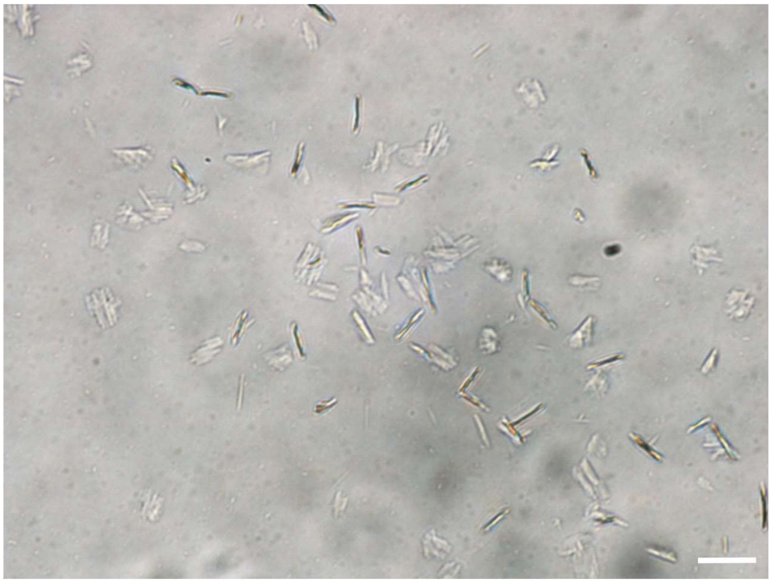

Supplementary Figure 5. Macroscopic GI R387A crystals: Polarized light microscopy image of GI R387A crystals (10min, Scalebar: 5µm).

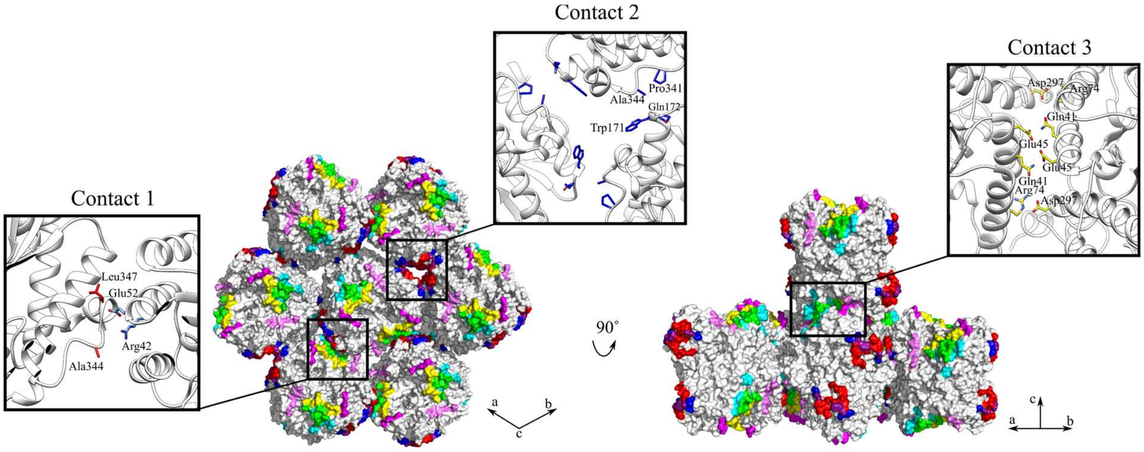

Supplementary Figure 6. Crystal lattice contacts of GI in the trigonal space group H32: Surface rendering of the nearest neighbors and crystal lattice contacts of GI in the trigonal space group H32: residues that partake in crystal packing contacts are colored according to the color scheme shown in Supplementary Table 1. Note that some residues are involved in two different contacts; the corresponding regional overlaps between neighboring patches are color coded according to patch H1a (red), H2 (blue), H1a/H2 overlap (purple); H1b (cyan), H3 (yellow), H1b/H3 overlap (green).

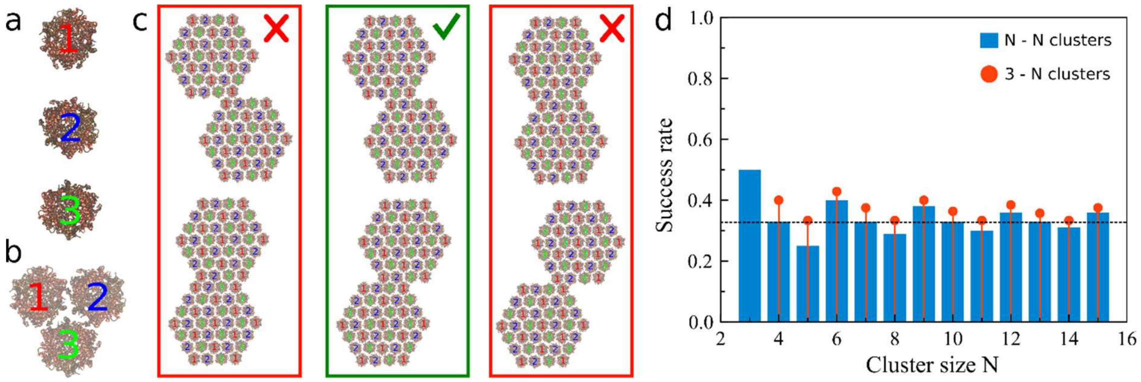

Supplementary Figure 7. Molecular orientations of a GI tetramer and docking probabilities: (a) The three different molecular orientations of a GI tetramer within the (001) plane of the H32 space group; (b) local arrangement around the three fold screw axis; (c) possible configurations for two clusters with facets of size N=4 to interact laterally: 2 out of 6 configurations lead to succesfull bond formation; (d) probabilities for succesful docking derived from the configurations in (b) as a function of cluster size, where N indicates the number of molecules for each facet: N-N denotes interactions between two clusters of size N, whereas 3-N corresponds to docking of a cluster of size 3, with clusters of varying size N.

Supplementary Note: Lattice contact analysis of H32 R387A crystals

Here we evaluate the molecular lattice contacts within the H32 space group. As we do not have crystallographic data for macroscopic R387A H32 crystals (due to twinning), we base our analysis on diffraction data that was collected for H32 crystals that were grown from a closely related point mutant, dubbed S171W GI. This approach is valid because there is good agreement between the unit cells derived from X-ray data for S171W (Supplementary Table 2) and those derived from the cryoEM data for R387A (see Supplementary Fig.2). In the H32 space group, each GI molecule has eight nearest neighbors (Supplementary Fig.6). Six of these lie within the (001) plane and involve the formation of two different types of lattice contacts, C1 and C2 (Supplementary Table 1). The remaining two neighbors are formed along the c-axis each making the same C3 contact with the central molecule. These crystallographic contacts (C1, C2 and C3) are formed by the recognition of different patches on the molecular surface of GI. C1 involves the burial of patch H1a on the central reference molecule and H1b on the corresponding symmetry mate. C2, however, is formed by the self-recognition of a single surface patch, denoted as H2. This means that two identical regions on the surfaces of two symmetry related copies interface to produce contact C2. Similarly, the contact C3 comprises two identical copies of patch H3, but also the interfacing of two minor patches H3a and H3b. It is also important to note that each GI molecule is a homo-tetramer with C2 symmetry, which means that there are multiple copies of each patch on a single tetramer. The H32 contacts described above are unique for this space group, there is little to no overlap in terms of the associated surface patches (I1a and I1b) with the contacts that exist in I222 crystals. This may explain why we do not see any GI H32 nanocrystals that serve as an epitaxial platform for the formation of an I222 lattice, and indicates that nucleation of each polymorph occurs independent of the other.

Supplementary Table 1: Lattice-contact analysis of GI in space group H32: two contacts (C1 and C2) are made within the (001) plane, and one contact (C3) is made along the *c*-axis. The columns ‘H’ and ‘S’ denote the number of hydrogen bonds and salt-bridges involved in the respective contacts. The ΔASA is the change in surface accessible area in $Å^2$ of the surface patches that are involved in the contact formation. Residues that are involved in a hydrogen bond or salt bridge with a nearest-neighbor residue are shown in bold and red, respectively. Residues highlighted in purple or green are shared between different patches (purple: H1a and H2; green: H1b and H3).

Contact	H	S	Patch	Symmetry	ΔASA	Residues
C1	2	0	H1a	X,Y,Z	390	K159, L164, E167, T170, W171, E207, R208, **A344**, D345, G346, **L347**, Q348, A349
			H1b	-Y-1,X-Y-1,Z	384	Q4, P5, E38, Q41, **R42**, A44, **E45**, D81, T82, D297, W300
C2	0	0	H2	-X,-X+Y,-Z	59	W171, Q172, G173, P341, A344
C3	2	3	H3	X-Y-1/3,-Y-2/3,-Z+1/3	350	R32, D35, V37, E38, **Q41**, R42, **E45**, R74, D295, F296, D297
			H3a	X,Y,Z	88	E67, E70, K73
			H3b	-X+Y-1/3,-X-2/3,Z+1/3	83	E328, A332, R334, A364, R368

Supplementary Table 2: X-ray crystallography: data collection and refinement statistics.

	**H32 (S171W)**
Resolution (Å)[a]	2.13 (2.13–2.19)
Space group	*H32*
Cell dimensions (Å; a, b, c); angle (°; α, β, γ)	132.9, 132.9, 234.9; 90, 90, 120
Total/Unique reflections	605,407/ 44,769
Completeness (%)[a]	99.9 (99.5)
R_{merge}[a]	0.145 (1.49)
R_{pim}[a]	0.059 (0.620)
I/σ(I)[a]	11.2 (1.7)
$CC_{1/2}$	0.998 (0.562)
Multiplicity	13.5 (12.9)
R_{cryst}	19.60%
R_{free}	22.80%
Rmsd in bond lengths (Å)	0.012
Rmsd in bond angles (°)	1.80
B- factor statistics (Å^2)	
Protein all atoms	36.81
Protein main chain atoms	34.81
Protein side chain atoms	38.86
Mn ions	78.96
Solvent atoms	38.93
Ramachandran statistics (Molprobity)	
Favoured	95.45%
Outliers	0.26%

[a]Values in parentheses refer to the highest resolution shell.
